# Dominant-negative heterozygous mutations in AIRE confer diverse autoimmune phenotypes

**DOI:** 10.1016/j.isci.2023.106818

**Published:** 2023-05-05

**Authors:** Bergithe E. Oftedal, Kristian Assing, Safa Baris, Stephanie L. Safgren, Isik S. Johansen, Marianne Antonius Jakobsen, Dusica Babovic-Vuksanovic, Katherine Agre, Eric W. Klee, Emina Majcic, Elise M.N. Ferré, Monica M. Schmitt, Tom DiMaggio, Lindsey B. Rosen, Muhammad Obaidur Rahman, Dionisios Chrysis, Aristeidis Giannakopoulos, Maria Tallon Garcia, Luis Ignacio González-Granado, Katherine Stanley, Jessica Galant-Swafford, Pim Suwannarat, Isabelle Meyts, Michail S. Lionakis, Eystein S. Husebye

**Affiliations:** 1 Department of Clinical Science, University of Bergen and Department of Medicine, Haukeland University Hospital, Bergen, Norway; 2 Department of Clinical Immunology, Odense University Hospital, Odense, Denmark; 3 Marmara University, Faculty of Medicine, Pediatric Allergy and Immunology, Istanbul, Turkey; 4 Istanbul Jeffrey Modell Diagnostic and Research Center for Primary Immunodeficiencies, Istanbul, Turkey; 5 Center for Individualized Medicine, Department of Quantitative Health Sciences, Mayo Clinic, Rochester, MN, USA; 6 Department of Infectious Diseases, Odense University Hospital, Odense, Denmark; 7 Department of Clinical Genomics, Mayo Clinic, Rochester, MN, USA; 8 Invitae, San Francisco, CA, USA; 9 Mayo Clinic, Department of Quantitative Health Sciences, Rochester, MN, USA; 10 Laboratory of Clinical Immunology & Microbiology, National Institute of Allergy & Infectious Diseases (NIAID), National Institutes of Health (NIH), Bethesda, MD, USA; 11 Department of Pediatrics, Division of Pediatric Endocrinology, Medical School, University of Patras, Rion, Greece; 12 Pediatric Hematology and Oncology Department, Hospital Álvaro Cunqueiro, Vigo, Spain; 13 Unidad de Inmunodeficiencias, Pediatría, Instituto de Investigación Hospital 12 de Octubre, Facultad de Medicina, Universidad Complutense, Madrid, Spain; 14 Mid-Atlantic Permanente Medical Group, Kaiser Permanente MidAtlantic, Rockville, MD, USA; 15 Division of Allergy & Clinical Immunology, National Jewish Health, Denver, CO, USA; 16 Department of Pediatrics, University Hospital Leuven, Laboratory for Inborn Errors of Immunity, Department of Microbiology Immunology and Transplantation, KU Leuven, Leuven, Belgium

**Keywords:** Human Genetics, Molecular genetics

## Abstract

Autoimmune polyendocrine syndrome type 1 (APS-1) is an autosomal recessive disease characterized by severe and childhood onset organ-specific autoimmunity caused by mutations in the autoimmune regulator (*AIRE*) gene. More recently, dominant-negative mutations within the PHD1, PHD2, and SAND domains have been associated with an incompletely penetrant milder phenotype with later onset familial clustering, often masquerading as organ-specific autoimmunity. Patients with immunodeficiencies or autoimmunity where genetic analyses revealed heterozygous *AIRE* mutations were included in the study and the dominant-negative effects of the *AIRE* mutations were functionally assessed *in vitro*. We here report additional families with phenotypes ranging from immunodeficiency, enteropathy, and vitiligo to asymptomatic carrier status. APS-1-specific autoantibodies can hint to the presence of these pathogenic AIRE variants although their absence does not rule out their presence. Our findings suggest functional studies of heterozygous AIRE variants and close follow-up of identified individuals and their families.

## Highlights

• Dominant-negative mutations in AIRE cause autoimmune disease
• Characterized by immunodeficiency, enteropathy, and vitiligo
• Functional studies of heterozygous AIRE variants are important
• Close follow-up of identified individuals and their families is recommended

## Graphical abstract

**Figure:**
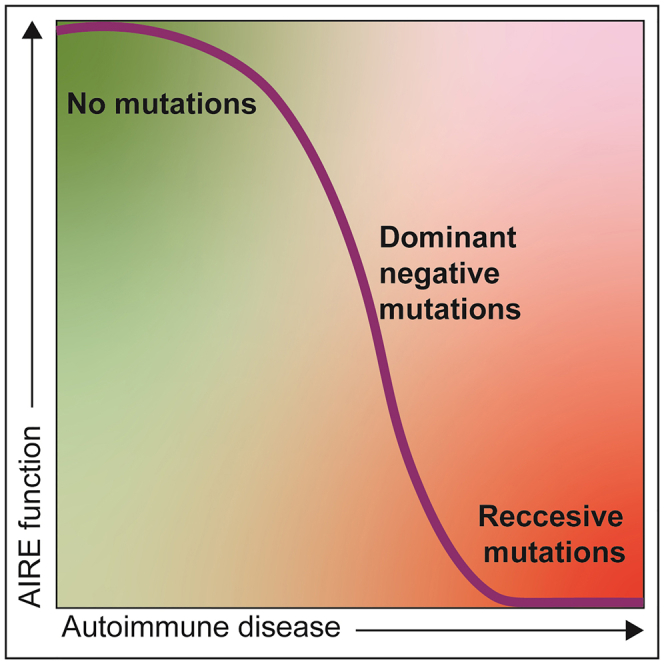

## Introduction

The autoimmune regulator (AIRE) is essential for the establishment of central immunological tolerance. AIRE supports the expression and presentation of tissue-restricted antigens (TRAs) for developing T cells in the thymus,^1^ promoting removal of autoreactive T cells from the repertoire by negative selection. Furthermore, AIRE promotes the expression of natural T regulatory cells capable of suppressing autoreactive T cells in the periphery.^2^ Patients with autoimmune polyendocrine syndrome type 1 (APS-1) (OMIM: 240300) illustrate the consequences of loss of AIRE. They develop autoimmunity in multiple organs, with primary adrenal insufficiency (Addison's disease), hypoparathyroidism, and chronic mucocutaneous candidiasis (CMC) as main components.^3^ Enamel hypoplasia, alopecia, vitiligo, pneumonitis, hepatitis, autoimmune gastritis, and enteropathy are also common manifestations.

With an estimated prevalence of 1:100 000, APS-1 is a rare disease.^3^^,^^4^^,^^5^ Diagnosis is based on clinical presentation (two of three main components) or disease-causing *AIRE* mutations. Type I interferon autoantibodies are present in 98% of patients and have proven effective as screening tools.^3^^,^^6^ However, more subtle reduction in AIRE function can also predispose to autoimmunity. Patients harboring dominant-negative heterozygous mutations in AIRE, especially within the plant homeodomain 1 (PHD-1) domain, present with a milder phenotype and later onset with a propensity for pernicious anemia and vitiligo.^7^ In mouse models replicating some of these mutations, the underlying mechanism was shown to be abrogated multimerization of AIRE, hindering its binding to DNA.^8^ Interestingly, these variants are relatively common in the general population with an estimated prevalence of up to 1:1000.^7^ Genome-wide association studies of autoimmune Addison's disease and pernicious anemia recently found a strong association to another AIRE variant located in the PHD2 domain (p.R471C), indicating that subtle changes in AIRE’s function predispose to autoimmunity against the adrenal cortex, gastric mucosa, and pancreas.^9^^,^^10^^,^^11^

Here, we expand the spectrum of variants in AIRE’s PHD domains associated with autoimmune diseases, describing patients harboring heterozygous mutations within the PHD1 and PHD2 domains of AIRE. The genetic and phenotypic characterization of patients and their families emphasize AIRE’s critical role in central immunological tolerance induction and indicates a correlation between autoimmune disease and AIRE function.

## Results

### Patient identification and genetic variants in AIRE

From 2016, eight heterozygous point mutations were identified within the PHD1 and PHD2 domains of AIRE in 11 patients with symptoms of autoimmunity or immunodeficiencies. These were identified while undergoing whole-exome or genome sequencing at their treating hospitals, or by targeted *AIRE* sequencing of patients in the Norwegian Registry of Organ-Specific Autoimmune Diseases (Table 1). Investigations of the index patients’ families identified nine additional heterozygous carriers.

**Table 1:** Sequencing of AIRE

Family/patient	Sequencing method	Country
I	WES was performed by GeneDx identifying the AIRE mutation. WES was repeated at NIH identifying the same AIRE variant. For WES at NIH, libraries were generated using the TruSeq DNA Sample Prep Kit (Illumina) and exome-enriched libraries were sequenced on the HiSeq 2500 instrument, according to the manufacturer’s protocol (Illumina).	USA
II	NGS using a customized panel of 192 PID genes (Ampliseq, Life Technologies)	Spain
III	Exome sequencing	Belgium
IV	WES sequencing was performed by Illumina (Illumina NextSeq-500). The pathological variant in the *AIRE* gene was confirmed by Sanger sequencing	USA/Greece
V	WES sequencing was done by GeneDx Clinical Exome Sequencing Analysis (XomeDxPlus). Mean depth of coverage was 153x, 98.7 of the genome was covered by at least 10x. There were no clinically relevant deletions or duplications identified on the WES analysis.	USA
VI	Next-generation sequencing using a genetic panel	Turkey
VII	The Invitae primary immunodeficiency panel (https://www.invitae.com/en/providers/test-catalog/test-08100) found the variant, which was also identified in by WES performed at NIH (methods as above for patient I).	USA
VIII	Sanger sequencing of AIRE	Norway
IX	Clinical testing through Prevention Genetics included sequencing and copy number variant analysis	England
X	The Invitae primary immunodeficiency panel (https://www.invitae.com/en/providers/test-catalog/test-08100) found the variant, which was then identified in the WES that we performed at NIH (methods as above for patients I and VII).	USA
XI	NGS using a customized AmpliSeq panel covering 291 PID genes	Denmark

**Figure 1:**
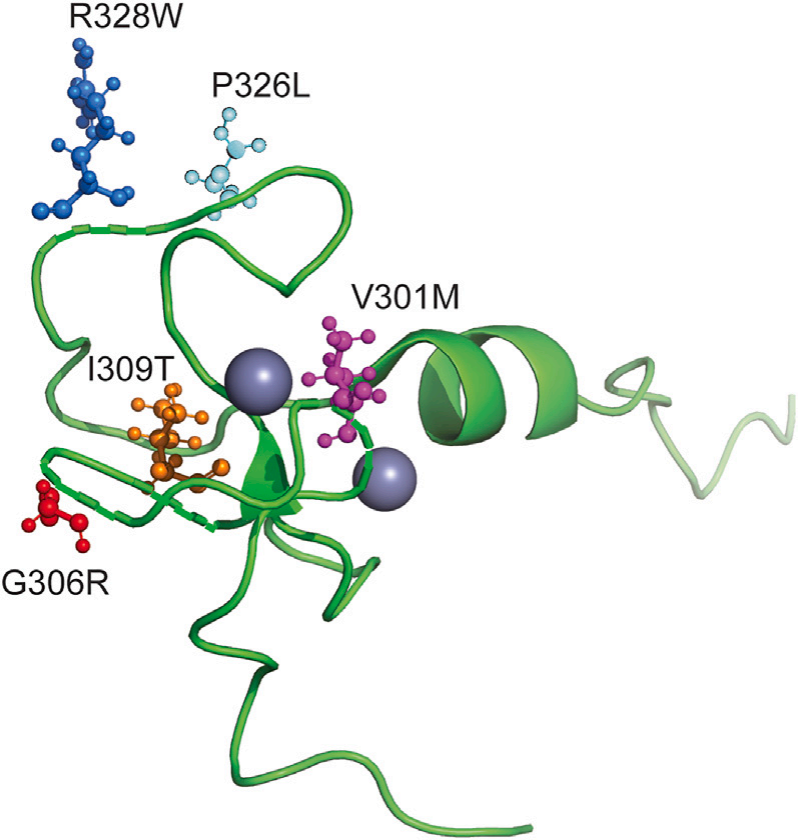
AIRE’s structure and the investigated mutations within the PHD1 domain The structure of AIRE’s PHD1 domain with the investigated amino acid changes shown as ball and stick figures; p.V301M purple, p.G306R red, p.I309T orange, p.R32W blue. The original amino acids are shown in the figure. The figure was made using The PyMOL Molecular Graphics System, Version 2.0 Schrödinger, LLC.

WES: Whole-exome sequencing, NGS: Next-generation sequencing.

c.901G>A (p.V301M), c.916G>A (p.G306R), c.926T>C (p.I309T), c.977C>T (p.P326L), and c.982C>T (p.328W) were located within PHD1 (Figure 1), while c.1102C>G (p.368A) and c.1235 C>T (S412L) variants were located between PHD1 and PHD2. A heterozygous c.1399G>C (p.G467R) change was found in PHD2. An overview of the AIRE mutations described is given in Table 2. Of these variants, c.926T>C p.I309T had not been reported previously with this particular amino acid change in the publicly available databases. The others have been reported with varying allele frequencies, c.901G>A (p.V301M) being the most common with an allele frequency of 1.06e-3 (Tables 3 and S1).

**Table 2:** Kindreds and the *AIRE* mutations described

Number of mutations	Total	8
	Dominant negative	7
	Not dominant negative	1
Location in AIRE, dominant-negative mutations	PHD1	5
	PHD2	1
	Other	1
Number of kindreds (persons)		11, (25)
Family members with mutation		20
	With symptoms	18
Family members without mutations		5
	With symptoms	3

**Table 3:** Population frequencies of the AIRE variants

AIRE variant	Protein change	Domain	gnomAD database allele frequency	Ensmble MAF
c.901G>A	p.V301M	PHD1	1.06e-3	<0.001
c.916G>A	p.G306R	PHD1	4.01e-6	–
c.926T>C	p.I309T	PHD1	Only changes to Valin or Methionine reported	
c.977C>T	p.P326L	PHD1	8.36e-6	–
c.982C>T	p.R328W	PHD1	2.08e-4	–
c.1102C>G	p.P368A	after PHD1	4.03e-6	–
c.1399G>C	p.G467R	PHD2	3.01e-5	<0.001

### Dominant-negative effect of AIRE variants

**Figure 2:**
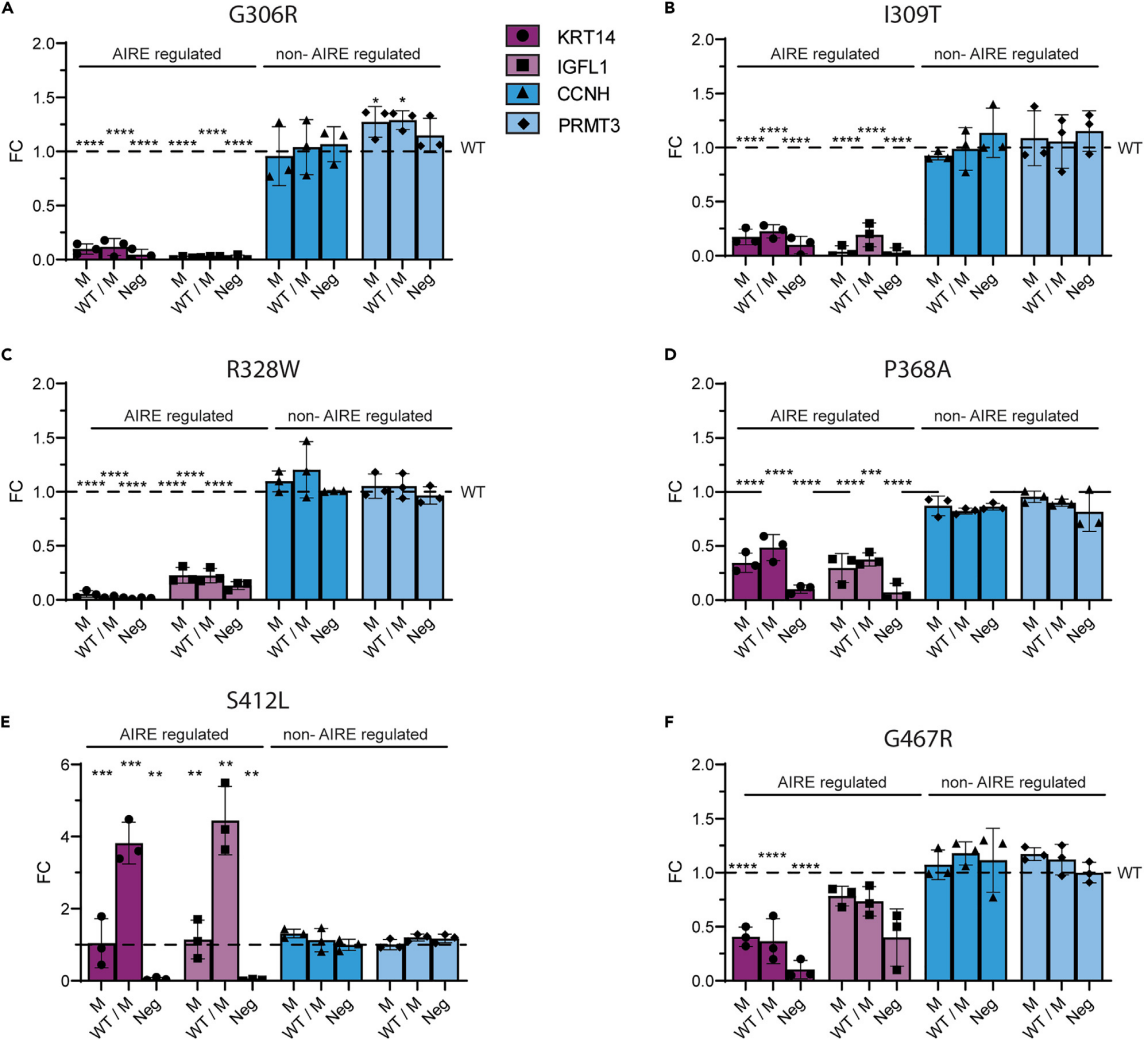
*In vitro* assay of AIRE-regulated genes measuring dominant capacity 4DC cells were transfected with plasmids containing AIRE with the different mutations (M) or equal amount of the mutation and wild-type (WT) AIRE. Expression of the AIRE-regulated genes KRT14 and IGFL1 (purple) and the non-AIRE-regulated genes CCNH and PRMT3 (blue) were used to determine transcriptional activity of AIRE. (A) the effect of the glycine (G) to arginine (R) change at amino acid position 306 in AIRE. (B and C) (B) the effect of an isoleucine (I) to a threonine (T) change at position 309, and (C) a change from arginine to tryptophan (W) at position 328. (D) A change from poline (P) to alanine (A) at position 368. (E and F) (E) A change from serine (S) to leucine (L) in the PHD2 domain at position 412 and (F) from glycine to arginine at amino acid position 467. Error bars represent standard error of the mean (SEM) of three separate experiments. ∗p < 0.05 and ∗∗p < 0.001 using Student's t-test comparing mutant and WT AIRE.

To confirm a dominant-negative effect, we used an in-house *in vitro* assay measuring the expression of AIRE-regulated genes upon co-transfecting cells with wild type and mutant AIRE plasmids.^7^ Seven out of the eight heterozygous mutations were shown to have a dominant-negative effect. We have previously reported the p.V301M and p.P326L variants as dominant negative^7^ while we here found p.G306R, I309T, and p.R328W within the PHD1 domain to diminish the expression of AIRE-regulated genes when co-transfected in a 1:1 ratio with wild-type AIRE (Figures 2A–2C). p.P368A, located between PHD1 and PHD2, had a dominant-negative effect (Figure 2D), while p.S412L did not (Figure 2E). Furthermore, p.G467R, located within the PHD2 domain, had a dominant-negative effect (Figure 2F).

### Clinical picture of patients with dominant-negative mutations within the AIRE PHD1 domain

The phenotypes ranged from no autoimmune manifestation to severe autoimmune disease accompanied by autoantibodies (Tables 4 and 5 and Figure S1). p.V301M, previously described as a dominant-negative mutation in humans *in vitro*, was found in three different individuals/families with a variable clinical presentation. In **family I**, the index patient (II-I) presented with anaphylaxis, angioedema, abdominal pain, migraine type headache, gastritis, intestinal dysfunction, CMC, food intolerance, and pernicious anemia. Several of these disease components are also common in the classical form of APS-1, in particular gastritis, pernicious anemia, and CMC. Pernicious anemia is commonly seen in other patients with PHD1 AIRE mutations. However, the patient was negative for type I interferon- and the Th17 cytokine-targeted autoantibodies tested. Her father (I-I) also harbored the heterozygous p.V301M mutation, presenting with symptoms of hives and heat intolerance, while her son (III-I) was diagnosed with urticaria pigmentosa since birth, severe diarrhea, and a pituitary adenoma. Of note, her daughter without this AIRE variant also had food intolerances with abdominal pain and diarrhea.

**Table 4:** Patients and their families

Family	Patient	Sex, relation	Place of birth	AIRE mutation	Protein change	Disease components	Autoantibodies	AIRE mutation previously described
I	I-I	Father	USA	c.901G>A	p.V301M	Hives and heat intolerance	ND	^7^^,^^8^
I	I-II	Mother	USA	Nf	nf	Hypothyroidism	ND	
I	**II-I**	**Mother/Daughter**	USA	c.901G>A	p.V301M	Anaphylaxis, angioedema, abdominal pain, migraine type headache, gastritis, intestinal dysfunction, CMC, food intolerance, and pernicious anemia	Negative^b^	
I	III-I	Son	USA	c.901G>A	p.V301M	Urticaria pigmentosa since birth, severe diarrhea, and pituitary adenoma	ND	
I	III-II	Daughter	USA	Nf	nf	Food intolerances with abdominal pain, diarrhea, and heartburn	ND	
II	I-I	Male	Spain	c.901G>A	p.V301M	Enteropathy	IFN-ω, IL-22, otherwise negative^b^	
III	I-I	Female	Belgium	c.901G>A	p.V301M	Neutropenia, cheilitis angularis, and enteropathy	Negative^a^	
IV	I-I	Uncle	Greece	c.916G>A	p.G306R	No disease components	Negative^b^	New
IV	I-II	Mother	Greece	c.916G>A	p.G306R	No disease components	Negative^b^	
IV	**II-I**	**Daughter**	Greece	c.916G>A	p.G306R	HP	Positive AAbs against IFN-ω, negative for^b^	
V	I-I	Male	USA	c.916G>A	p.G306R	Multifocal seizures, DM, pernicious anemia, deep set eyes, and short philtrum	Positive for GAD and 21OH, otherwise negative^b^	
VI	I-I	Father	Turkey	nf	nf	No disease components	ND	New
VI	I-II	Mother	Turkey	c.926T>C	p.I309T	Chronic fatigue, leukopenia, lymphopenia, and low B12	anti-thyroid peroxidase antibody, otherwise negative^a^	
VI	**II-I**	**Son**	Turkey	c.926T>C	p.I309T	V, CD, H, and short status	IFN-ω, 21-OH, TPH, otherwise negative^a^	
VII	I-I	Male	USA	c.977C>T	p.P326L	Immunodeficiency, recurrent oropharyngeal candidiasis, migraines, and chronic diarrhea	Negative^b^	^7^
VIII	I-I	Father	Norway	nf	nf	No disease components	ND	New, but p.R328Q was described in^7^
VIII	I-II	Mother	Norway	c.982C>T	p.R328W	V, DM, and PS	GAD, otherwise negative^a^	
VIII	**II-I**	**Daughter**	Norway	c.982C>T	p.R328W	V, CD, and As	Negative^a^	
VIII	II-II	Daughter	Norway	nf	nf	V and UC	Negative^a^	
VIII	II-III	Son	Norway	c.982C>T	p.R328W	V, As, and B-12 def	Negative^a^	
IX	I-I	Mother	England	c.982C>T	p.R328W	Transient B-12 def	Negative^b^	
IX	**II-I**	**Daughter**	England	c.982C>T	p.R328W	Congenital diarrhea with positive stool reducing substances	Negative IFN-ω^b^	
X	I-I	Mother	USA	c.1102C>G	p.P368A	Vaginal candidiasis, and onychomycosis	ND	New
X	**II-I**	**Daughter**	USA	c.1102C>G	p.P368A	Migraine type headache, chronic constipation, poor appetite, recurrent fever, proteinuria, postural orthostatic tachycardia syndrome, atypical compound melanocytic nevi (BRAFV600E mutation with retained BAP-1 expression), and elevated hemoglobin A1C at 5.8% and fasting glucose of 104.	Positive for IL17A^b^	
XI	I-I	Male	Denmark	c.1399G>C	p.G467R	Immunodeficiency	Positive IgM RA, 21-OH, SSC, anti-GPIa-IIa, anti-GPIIb-IIIa, anti-GPIb-IX, anti-GPIV, otherwise negative^a^	New

HP, Hypoparathyroidism; V, Vitiligo, CD; Celiac disease; As, Asthma; B-12 def, B-12 deficiency; DM, Diabetes mellitus, PS, Psoriasis, H, Autoimmune hepatitis. Aspln, Asplenia; Nd, Not done, nf, not found.

a Autoantibodies against the following antigens were tested: 21-OH, GAD, SCC, 12-OH, AADC, TPH, TH, IFN-w, Nalp-5, IL-17A, and IL-22.

b Autoantibodies against the following antigens were tested: IFNγ, IFNα, IFNβ, IFN-ω, GM-CSF, IL-1a, IL-12p70, IL-17A, IL-17F, IL-22, and IL-23.

**Table 5:** Patient summary

	All patients with mutations (n = 20)	Index patients (n = 11)
Age at onset (mean (range))	na	13,3 (0–68)
Age at diagnosis (mean (range))	na	25,6 (1–72)
Diagnostic delay (mean (range))	na	12,3 (1–35)
Enteropathy, gastritis, UC (number, (%))	8 (40)	5 (45.5)
Vitiligo (number, (%))	4 (20)	2 (18.2)
B12 deficiency (number, (%))	3 (15)	0 (0)
Pernicious anemia (number, (%))	2 (10)	2 (18.2)
Diabetes (number, (%))	2 (10)	1 (9.1)
Immunodeficiency (number, (%))	2 (10)	2 (18.2)
No manifestations (number, (%))	2 (10)	0 (0)
Cytokine antibodies (number, (%))	4 (20)	4 (36.4)
Organ-specific antibodies (number, (%))	5 (25)	3 (27.3)
Female/male ratio	11:9	6:5

The young boy in **family II** with p.V301M was diagnosed with enteropathy. Interestingly, he was positive for autoantibodies against both interferon (IFN)-ω and interleukin (IL)-22, autoantibodies which are found in almost all patients with classical APS-1. In **family III**, the index patient with p.V301M presented at the age of 3 years and 5 months with a history of recurrent upper respiratory tract infections and skin infections since birth, with a severe course of cellulitis. She also had angular cheilitis and chronic gingivitis. Upon physical examination, she was at −2 SD for weight and height. Blood analysis revealed a severe chronic intermittent neutropenia of <500 mcl. No anti-neutrophil antibodies were found but bone marrow and peripheral blood analysis was in line with a potential autoimmune neutropenia. Other immunological investigations, including immunoglobulin levels, immunophenotyping, and STAT1 phosphorylation, were all normal. Skeletal survey and the amylase pattern were normal as well. Bone mineral content was normal for a six-year-old individual. She was started on filgrastim 5 μg/kg three times per week, which improved her condition significantly. At 11 years of age, gastrointestinal endoscopy revealed microscopic colitis, which was successfully treated with mesalazine. More recently, she has been suffering from intermittent arthritis.

The heterozygous AIRE mutation p.G306R was found in two non-related patients, a girl from Greece and a boy from the United States. The index patient in **family IV** (II-I) was diagnosed with hypoparathyroidism and was also positive for autoantibodies against IFN-ω. Her mother (I-II) and uncle (II-I) also harbored the same mutation but had neither autoimmune clinical manifestations nor autoantibodies. **Patient V** is a 14-year-old male patient referred to the Mayo Clinic for evaluation of refractory status epilepticus and type 1 diabetes. During pregnancy, his mother experienced hypertension, but the patient was born at term without neonatal complications. Early psychomotor development was normal. Multifocal seizures started at age 5 years of age and were often severe requiring hospitalization. When he was 14 years old, he was diagnosed with type 1 diabetes that is currently well controlled. Several family members have autoimmune disorders including thyroid disorders. The patient has pernicious anemia and high GAD65 antibodies consistent with a type 1 diabetes, but also with autoimmune epilepsy. The patient has deep set eyes and short philtrum but is otherwise without dysmorphic features. Whole-exome sequencing identified c.916G>A, a *de novo* AIRE variant (p.G306R) in exon 8 as the mother and father did not carry this variant.

Three amino acids upstream, a heterozygous p.I309T variant was found in an 18-year-old male patient (II-I) (**family VI**) with multiple autoimmune presentations and recurring fever. At 8 years of age, endoscopy and intestinal biopsy revealed increased intra-epithelial lymphocytes, crypt hyperplasia, and blunted villi, although anti-transglutaminase antibodies were negative. He was started on gluten-free diet. At 13 years of age, liver biopsy revealed autoimmune hepatitis successfully treated with glucocorticoids and azathioprine.^12^ He had growth retardation (weight and height <3p), intermittent neutropenia (970–1500/mm^3^), and lymphopenia (560–2460/mm^3^), but bone marrow aspiration was normal. At 16 years of age, he was started on anti-tuberculous therapy because of pulmonary infiltrations and high purified protein derivative as 20 mm. Autoantibodies were positive for anti-parietal antigen, anti-IFN-ω, anti-21OH, and anti-TPH. He also had low IgG (882 mg/dL) and IgM (74 mg/dL) with normal IgA and IgE. Extensive immunophenotyping revealed CD4 lymphopenia (455/mm^3^), CD8 lymphopenia (188/mm^3^), and CD16 + 56 lymphopenia (23/mm^3^). He also had low levels of naive CD4+T cells (29%) and recent thymic emigrants (20%). During follow-up at 16 years of age, he developed difficulty walking and laboratory findings showed high creatinine kinase level at 596 IU/mL. His electromyography was compatible with proximal myopathy. Muscle biopsy demonstrated atrophy with degeneration with minimal inflammatory component. Systemic corticosteroids, plasmapheresis, and high-dose intravenous immunoglobulins were given without response on the myopathy. Currently, the patient continues to receive antimicrobial and antifungal prophylaxis. His mother (53-year-old) had the same heterozygous variant and chronic fatigue without other organ-specific autoimmune manifestations. She had leukopenia (3300/mm^3^) and lymphopenia (900/mm^3^), very low B12 (67 ng/dL), and high anti-thyroid peroxidase antibody (628 U/ml) with normal thyroid function tests.

**Patient VII** had a heterozygous p.P326L variant. He presented at age 28 for recurrent sinus infections and oropharyngeal candidiasis starting at age 9. He required antifungal prophylaxis for each course of antibiotics he received starting in childhood. He also had chronic diarrhea with abdominal pain and migraines. Aeroallergen skin testing was negative and the total IgE was 112kU/L. He had normal thyroid hormone (TSH 1.14mIU/L) and hemoglobin A1c (5.4%) levels. No other autoantibodies were identified. He had elevated liver enzymes (AST 72U/L, ALT 97U/L) with imaging suggesting non-alcoholic fatty liver disease. Anti-mitochondrial and anti-smooth muscle antibodies were negative. Endoscopy suggested gastric reflux disease and showed mild gastritis. No reduction in neuroendocrine cells was seen and vitamin B12 was normal. Immunologically, he had normal quantitative IgG, mildly low total CD4 T lymphocyte count (423 cells/μL [ref. 430–1800 cells/μL]), and low CD27+IgD-IgM-class-switched memory B cells 2 cells/μL (11–61 cells/μL) with waning vaccination titers to *Streptococus pneumoniae* (>1.3 μg/mL for IgG to 14/23 serotypes 1 year post vaccination, 22/23 6 weeks post vaccination). He had normal percentages of Th1 (IFNγ+) and Th17 (IL-17+) T cells. In addition to a more aggressive intranasal anti-inflammatory and rising regimen, the patient was started on a trial of immune globulin replacement therapy for specific antibody deficiency which has helped reduce the frequency of sinus infections.

In **family VIII**, a heterozygous p.R328W change in AIRE was found in a mother and two children. They all had vitiligo, as did the sibling without the p.P328W variant. However, while the carriers of p.R328W had severe childhood onset vitiligo, the non-carriers had mild adult-onset vitiligo. The same variant was also seen in **family IX** where the index patient (II-I) had a history of loose, frequent stools since birth. This was associated with weight loss at around 3 months of age leading to additional evaluation. Stool alpha-1 antitrypsin and pancreatic elastase-1 were both normal. Stool reducing substances were positive (3+) on several occasions. Genetic testing identified heterozygosity for the p.R328W variant in AIRE. Her weight improved after addition of a hypoallergenic baby formula to her diet. Parathyroid, hepatic, and thyroid functions were normal. Her mother (I-I) has a history of transient B12 deficiency during her early 20s that resolved. She also had a history of abnormal glucose tolerance during pregnancy, eczema, asthma, and food allergy. Targeted variant testing revealed heterozygosity for the p.R328W variant. The index patient’s maternal grandmother tested negative for the variant while the index patient’s father is not available for testing. Her parathyroid hormone, calcium, vitamin B12, and blood counts were all normal at the latest examination.

In **family X**, a p.P368A mutation was found in the mother and a daughter (**Patient II-I**) with a broad clinical picture, ranging from migraine type headache, chronic constipation, poor appetite, recurrent fever, proteinuria, postural orthostatic tachycardia syndrome, atypical compound melanocytic nevi (BRAFV600E mutation with retained BAP-1 expression), and to elevated hemoglobin A1C at 5.8% and fasting glucose of 104. She was also positive for autoantibodies against IL-17A, often found in patients with APS-1. The patient’s mother reports vaginal candidiasis and onychomycosis beginning in childhood with significantly reduced frequencies as an adult.

**Patient XI,** a Danish male, presented with disseminated *Mycobacterium kansasii* infection with multiple (including liver) granulomas, abscess formation, and purulent arthritis at age 68 years. Autoantibodies directed against IFN-γ, IL-6, IL-1α, IL-17F, TNF-α, and IFN-ω were absent. A concomitant bone marrow biopsy showed granuloma formation, but no signs of malignancy. Four years earlier, the patient was diagnosed with immune thrombocytopenic purpurea and leukopenia (Table 4), subsequently also with hypogammaglobulinemia, the latter fulfilling the criteria for common variable immune deficiency (total IgG <4.5 g/L) leading to immunoglobulin substitution. Immunological work-up revealed total absence of B cells (0% of lymphocytes) and severely reduced CD4^+^ T cell numbers: 0.05 x 10^9^/L (normal range: 0.3–1.7 x 10^9^/L), the latter consisting mostly of CD45R0+ memory cells (98% of CD4^+^ T cells). A high degree of T cell activation was also recorded (HLA-DR+: 79% of CD3^+^ T cells). The CD8^+^ T cell concentrations were normal, but peripheral γδ CD3^+^ T cells were not detectable, and a test for human immunodeficiency virus was negative. The patient did not display classical APS-1 symptoms, nor vitiligo. Anti-nuclear antibodies were negative but IgM rheumatoid factor (titer 44), 21OH, and SSC autoantibodies were positive (Table 4). The patient had normal serum potassium and sodium and a normal morning plasma cortisol (292 nmol/L). Within the last two years, the patient was diagnosed and treated for a B cell lymphoma, which is now in full remission. Next-generation sequencing revealed a missense mutation in AIRE c.1399G>C (p.G467R) in the PHD2 domain. The variant is situated in a well-defined tertiary structure of PHD2, spanning the residues R433-S476.^13^ The background frequency of this mutation is 0.04% and Universal Mutation Database prediction classified it as disruptive.

## Discussion

Here, we report 20 individuals from 11 kindreds with dominant heterozygous mutations in AIRE located within or close to the PHD1 and PHD2 domains, all with dominant-negative effect *in vitro*. Enteropathy was a common symptom among the patients, where several also presenting with autoantibodies against IFN-ω.

Five variants were found in the PHD1 domain. The p.V301M variant was previously reported in a mother and her daughter where the mother presented with autoimmune Addison's disease, autoimmune thyroiditis, premature ovarian insufficiency, and autoantibodies targeting 21-hydroxylase, AADC, and IL-17F. Her daughter only had autoantibodies against IL-17F as a sign of autoimmunity.^7^ We here report five additional patients from three families with heterozygous p.V301M and APS-1 manifestations such as gastritis, CMC, pernicious anemia, and enteropathy. Some had autoantibodies against IFN-ω and IL-22, seen in almost all patients with APS-1. However, the corresponding mutation p.V303M in mice did not significantly alter the transcriptome of TRAs in mTECs or the development of regulatory T cells, hence questioning the dominancy of this mutation or highlighting the species background.^8^ Interestingly, the previously published *in vitro* assay measuring dominancy showed a variable pattern of dominancy in the AIRE-regulated genes tested, suggesting a milder effect of this variant.^7^ Furthermore, since V301M is relatively frequent, we would expect to see this mutation in patients with classical APS-1, but we do not. This indicates that p.V301M predisposes for other autoimmune manifestations than those typically associated with APS-1. Yet, p.V301M could work in concert with other genes and environmental triggers to elicit autoimmune manifestations.

Interestingly, several disease component and autoantibodies typically seen in classical APS-1 were observed, like autoantibodies against IL-22 and IFN-ω in patients with the p.V301M change. The previously unreported p.G306R heterozygote variant was found in two families, where hypoparathyroidism, pernicious anemia, and vitiligo as well as autoantibodies against IFN-ω were found. Vitiligo and autoantibodies against IFN-ω and 21-OH was also found in a boy from Turkey harboring a heterozygous p.I309T change. The p.R328W was found to be dominant negative in two families with manifestations such as vitiligo and B12 deficiency. A different change at the same position, an arginine to a glutamine, was previously reported as dominant negative in a patient with vitiligo and autoantibodies against glutamic acid decarboxylase and with gastric parietal cell antibodies.^7^ The young girl described herein had congenital diarrhea and will be interesting to follow-up with regards to the development of additional autoimmune manifestations.

**Figure 3:**
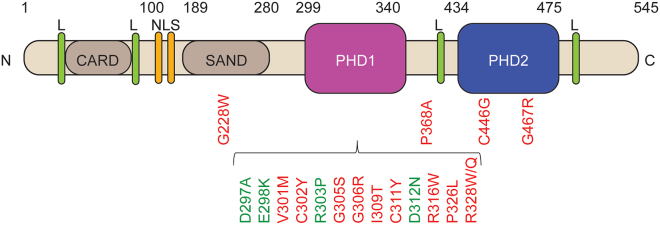
Overview of the heterozygous variants associated with a dominant-negative effect AIRE with the heterozygous variants described as dominant negative *in vitro*; the ones in red are found in patients, while the green variants are identified from variant databases and tested *in vitro*.

Moving out of the PHD1 domain, the results of the *in vitro* assays are not as clear. One patient with a heterozygous p.P368A variant had several APS-1-like components, such as hypoparathyroidism, enamel hypoplasia, and autoantibodies against IL-17A. She was diagnosed with immunodeficiency, as was the elderly male patient with yet another heterozygous variant in the PHD2 domain. He was also positive for several autoantibodies, among them against 21-OH and SCC, both common in patients with Addison's disease and APS-1. The mutations in the PHD2 domain have been less investigated, but recently a family with a p.C446G heterozygous variant was described, where one of the identified patients suffered from chronic diarrhea. The dominant-negative capacity of p.C446G was recently confirmed in a mouse model.^8^ An overview of all dominant-negative mutations described in AIRE is found in Figure 3.

Chronic diarrhea and enteropathy were frequently seen in these patients. In total, six of the patients reported here had such manifestations, and it was also reported in one of the patients with the p.C446G variant.^8^ As AIRE is suggested to play a role in the development of Tregs,^14^ these cells might contribute to enteropathy as this manifestation is common in patients with reduced T reg function. In the IPEX syndrome (Immune dysregulation, Polyendocrinopathy, Enteropathy, X-linked) caused by loss-of-function mutations in FOXP3 leading to loss of regulatory T cells is an apt example. These patients develop early-onset severe enteropathy, type 1 diabetes, and dermatitis.^15^ The thymic architecture is not disturbed in mice models of this disease, suggesting that the major role of Foxp3 is promotion of Treg cell differentiation within the T cell lineage.^16^ Also, patients with CTLA-4 insufficiency have compromised Treg function, leading to enteropathy and endocrinopathy. More than 50 heterozygous germline mutations have been reported, and incomplete penetrance and variable expressivity of genetic variants is common.^15^ Enteropathy has been reported together with immunodeficiency, autoimmunity, and alopecia as a result of thymic stromal malfunction in patients with tetratricopeptide repeat domain 7A (TTC7A) defects. Like AIRE, TTC7A is highly expressed in mTECs.^17^^,^^18^ Also, patients with thymoma can develop enteropathy^19^^,^^20^ in addition to autoimmune conditions, most commonly myasthenia gravis. They have defective epithelial expression of AIRE and a failure to generate Tregs.^21^ Interestingly, about 70% of patients with thymoma have autoantibodies neutralizing type I IFNs.^22^^,^^23^

The incomplete penetrance of these dominant-negative mutations resembles what has been found in other diseases, like hypercholesterolaemia^24^ and rhabdoid tumor predisposition syndrome.^25^ Factors like the action of unlinked modifier genes, epigenetic changes, or environmental factors may underlie this variable penetrance. We have not investigated such factors, and the small sample number will make their role challenging to determine. Looking to the mouse models of the dominant-negative mutations, recessive mutations (causing classical APS-1 in humans) result in a lack of expression of the AIRE protein, while the dominant mutations increase the expression of AIRE. Furthermore, AIRE’s ability to bind its own proximal enhancers is not altered, but they have reduced ability to induce changes in the accessibility to these loci.^8^ The mechanistic aspects of the new dominant AIRE mutations described herein requires further attention; however, the previously described mechanisms support the notion of a dosage dependency of AIRE, where the degree of accessibility vary in a stochastic manner. Interestingly, two single-nucleotide variants in AIRE were recently found to increase the risk of isolated autoimmune Addison's disease,^9^^,^^10^^,^^11^ supporting the concept that small changes in AIRE can predispose for autoimmune disease.

The classical APS-1 phenotype is rarely seen in patients with heterozygous dominant mutations although some have autoantibodies typical for APS-1. Instead, the clinical picture is diverse, with enteropathy, immunodeficiency, and vitiligo as common features. The fact that patients with the same mutation even in the same family have diverse or even no phenotype suggests that genetic, epigenetic, or environmental factors could alter the phenotype. As most of our patients are from Europe or America, a larger and more geographically diverse patient cohort is necessary to shed light on this. Looking at classical APS-1, AIRE mutations and phenotypes seem to be uncorrelated between countries and within families. Among four common AIRE mutations causing classical APS-1, two are found within certain populations; p.Y85C is common among Persian Jews while p.R139X is frequent among Sardinians.^26^^,^^27^ p.Y85C has been associated with less candidiasis. A 13-base pair deletion in exon 8 is found within British, Dutch, German, Finnish, and Norwegian patients with APS-1 and is also the most frequent AIRE mutation in India and America.^28^^,^^29^^,^^30^^,^^31^^,^^32^^,^^33^ p.R257X is commonly referred to as the major Finnish mutation but is also common among German, Swiss, British, Northern Italian, Russian, Polish, and Norwegian patients with APS-1.^33^ Interestingly, an AIRE mutation often seen in Italy, C322fsx372, is also found in India as the most common mutation among Muslim probands.^32^^,^^34^ There is no obvious correlation between mutations and phenotype, and large phenotypic variation among those with the same genotype, including siblings.

A close follow-up of patients with dominant-negative AIRE mutations and their families will reveal whether the risk of developing other and more severe autoimmune manifestations is increased. As whole-exome and genome sequencing are increasingly used in the diagnostic setting, we expect to see more heterozygous AIRE mutations in patients presenting with autoimmune disease and immunodeficiencies. Therefore, it is necessary to establish robust high-throughput *in vitro* assays and take advantage of data simulation programs to explore the impact of these mutations determining their pathogenic effects.

### Limitations of the study

There are certain limitations to our study. The major drawback is the lack of mechanistic insight into how these mutations reduce AIRE’s function and affects the presentation of tissue-restricted antigens in the thymus. Mouse models have been instrumental in answering these questions earlier^8^ but are expensive and time-consuming. Cell lines that stably express AIRE are lacking, hence overexpression of AIRE in cell lines is currently the best model but comes with limitations when reflecting a biological system. The emergence of thymic organoids will likely be a good model system to study human variants. Our study also lacks an unbiased search for patients, as no systematic identification of patients across hospitals has been done.

## STAR★Methods

### Key resources table

**Table:** 

REAGENT or RESOURCE	SOURCE	IDENTIFIER
**Biological samples**		

Patient derived blood serum	Local hospitals	
Patient derived blood DNA	Local hospitals	

**Critical commercial assays**		

QuikChange II Site-Directed Mutagenesis Kits	Agilent technologies	200523

**Experimental models: Cell lines**		

human thymic 4D6 epithelial cells	Gift from Professor Christophe Benoist, Harvard Medical School	

**Oligonucleotides**		

keratin 14 (KRT14)	Applied Biosystems	Hs00265033-m1
IGF-like family member 1	Applied Biosystems	Hs01651089-g1
AIRE	Applied Biosystems	Hs01102906-g1
beta_2_-microglobulin	Applied Biosystems	4333766
cyclin H (CCNH)	Applied Biosystems	Hs00236923_m1
protein arginine methyltransferase 3 (PRMT3)	Applied Biosystems	Hs00411605_m1
AIRE V301M_Fw	Sigma-Aldrich	GACGAGTGTGCCATGTGTCGGGACG
AIRE V301M_Rv	Sigma-Aldrich	CGTCCCCACACATGGCACACTCGTC
AIRE G306R_Fw	Sigma-Aldrich	GTCGGGACGGCAGGGAGCTCATCTG
AIRE G306R_Rv	Sigma-Aldrich	CAGATGAGCTCCCTGCCGTCCCGAC
AIRE I309M_Fw	Sigma-Aldrich	CGGGGAGCTCATGTGCTGTGACGGC
AIRE I309M_Rv	Sigma-Aldrich	GCCGTCACAGCACATGAGCTCCCCG
AIRE R326L_Fw	Sigma-Aldrich	GCCTGTCCCCTCTGCTCCGGGAGATC
AIRE R326L_Rv	Sigma-Aldrich	GATCTCCCGGAGCAGAGGGGACAGGC
AIRE R328W_Fw	Sigma-Aldrich	CTGTCCCCTCCGCTCTGGGAGATCCCCAGTG
AIRE R328W_Rv	Sigma-Aldrich	CACTGGGGATCTCCCAGAGCGGAGGGGACAG
AIRE P368A_Fw	Sigma-Aldrich	AGACCCCGCTCCCCGCGGGGCTTAGGT
AIRE P368A_Rv	Sigma-Aldrich	ACCTAAGCCCCGCGGGGAGCGGGGTCT
AIRE S412L_Fw	Sigma-Aldrich	AGGGCTGGACTCCTTGGCCCTGCACC
AIRE S412L_Rv	Sigma-Aldrich	GGTGCAGGGCCAAGGAGTCCAGCCCT
AIRE G467R_Fw	Sigma-Aldrich	ACCTCCCGGCCCAGGACGGGCCTGC
AIRE G467R_Rv	Sigma-Aldrich	GCAGGCCCGTCCTGGGCCGGGAGGT

**Recombinant DNA**		

Plasmid:		
svPoly	Gift from Dr. Ismo Ulmanen (Department of Molecular Medicine, National Public Health Institute)	

**Software and algorithms**		

PyMOL2.5	Schrodinger	https://pymol.org/
GraphPad Prism 9	Dotmatics	https://www.graphpad.com/
PrimerX	Bioinformatics.org	http://www.bioinformatics.org/primerx/

### Resource availability

#### Lead contact

Further information and requests for resources and reagents should be directed to and will be fulfilled by the lead contact, Bergithe E. Oftedal (Bergithe.oftedal@uib.no).

#### Materials availability

This study did not generate new unique reagents.

### Experimental model and subject details

#### Patients and ethical approvals

The patients were recruited between 2015-2021. Eleven patients/families with dominant heterozygous mutations in AIRE were included, in total 20 with AIRE variants (average age 25.6, range 1-72 years) and five relatives without (in total 10 males, 15 females). The families were from the United States (n=9), Greece (n=3), Spain (n=1), Turkey (n=3), Denmark (n=1), United Kingdom (n=2), Belgium (n=1), and Norway (n=5). They have been clinically characterized at their treating hospitals and the details of the AIRE sequencing are found in Table 1. All participants signed informed and written consent and the study was approved by the local ethics committees, including the Regional Committee for Medical and Health Research Ethics (approval number REK 2009/2055 and 2013/1504) (Norway and Turkey) and the BIOKID protocol approved by the ethics committee of Leuven. Publishing permission was obtained from the chairman of the Regional Committee on Health Research Ethics for Southern Denmark (S-20192000-48) (Denmark). At the Mayo Clinic, the patient was consented under IRB 12-009346, the patient/family provided consent to participate through National Institute of Health, but also signed consent to release information (HIPAA authorization) including for publication (England, Norway and Turkey), and the patients from the United States, Greece, and Spain enrolled onto a NIH IRB-approved research protocol (clinicaltrials.gov, NCT01386437). The study was performed in accordance with the Declaration of Helsinki.

#### Sequencing of AIRE

The details are found in Table 1. Most variants were identified by Sanger sequencing of AIRE. In patient V, exome sequencing was performed by GeneDx Clinical Exome Sequencing Analysis (XomeDxPlus). Mean depth of coverage was 153x; 98.7 of the genome was covered by at least 10x. There were no clinically relevant deletions or duplications identified on the whole exome sequencing (WES) analysis. Patient VIII had clinical testing through Prevention Genetics including sequencing and copy number variant analysis. Patient III also received WES.

#### Clinical analysis of autoantibodies

Autoantibodies against 21-hydroxylase (21OH), 17-α-hydroxylase (17OH), aromatic L-amino acid decarboxylase (AADC), glutamic acid decarboxylase 65-kDA isoform (GAD65), interferon-ω (IFN-ω), interleukin-17 (IL17), interleukin-22 (IL22), melanoma antigen B2 (MAGEB2), NACHT leucine-rich-repeat protein 5 (NALP5), protein disulfide isomerase-like testis expressed (PDILT), putative potassium channel regulator (KCNRG), side-chain-cleavage enzyme (SCC), sex determining region Y-box 10 (SOX10), transglutaminase 4 (TGM4), tryptophan hydroxylase 1 (TPH1), and tyrosine hydroxylase (TH) were screened for and assayed by radio-binding ligand assay as described previously.^35^^,^^36^^,^^37^ Parietal cell antigen (PCA) autoantibodies were assayed by ELISA (Euroimmun, Lübeck, Germany). Endocrinopathies were diagnosed based on both clinical and laboratory evidence of endocrine hypofunction as previously described.^38^ Autoantibodies against IFNγ, IFN-α, IFNβ, IFN-ω, GM-CSF, IL-1a, IL-12p70, IL-17A, IL-17F, IL-22, and IL-23 were evaluated in patients enrolled at the NIH using a particle-based approach, as previously described.^29^

### Method details

#### Plasmid construction

The plasmid svPoly containing human wild-type (WT) AIRE was a kind gift from Dr. Ismo Ulmanen (Department of Molecular Medicine, National Public Health Institute). The different mutations in AIRE were introduced by site-directed mutagenesis (QuikChange II Site-Directed Mutagenesis Kits [Agilent Technologies]) using primers designed by the web-based program PrimerX (http://www.bioinformatics.org/primerx/) (see key resources table for primers). All plasmids were verified by Sanger sequencing.

#### Cell culture, transfection, and RNA extraction

Human thymic 4D6 epithelial cells, a kind gift from Professor Christophe Benoist (Harvard Medical School),^39^^,^^40^ were cultured in RPMI 1640 (Sigma-Aldrich) containing 10% (v/v) fetal bovine serum, 10 mM HEPES buffer, 1% (v/v) non-essential amino acids (Lonza), 2 mM L-glutamine (Lonza), 100 U/ml penicillin, and 100 μg/ml streptomycin (Lonza) at 37°C with 5% CO2 in a humidified incubator. For transfection, cells were plated at a density of 5 × 105 cells per well in a 6-well plate and left overnight. Plasmids (3.3 μg) were added to a total volume of 157 μl supplemented RPMI 1640, added 8.3 μl Fugene HD transfection reagent (Promega Corporation) for 5 min at room temperature then added to the cells and incubated for 24 hr. RNA was extracted by RNeasy Mini Kit (QIAGEN) according to the manufacturers’ protocol, including in-column DNase treatment. cDNA was prepared from 1 μg of total RNA (High-Capacity RNA-to-cDNA Kit, Applied Biosystems). All transfections were repeated a minimum of three times.

#### Assay of AIRE-regulated genes

The ability of transfected AIRE plasmids to induce expression of selected AIRE-regulated genes and control genes were measured by quantitative PCR (qPCR) using the primers and probes (Applied Biosystems, California, US): keratin 14 (KRT14) (Hs00265033-m1),IGF-like family member 1 (IGFL1) (Hs01651089-g1), and AIRE (Hs01102906-g1 and Hs01102908-g1) using beta_2_-microglobulin (B2M) (4333766) as endogenous control and the AIRE-independent genes cyclin H (CCNH) (Hs00236923_m1) and protein arginine methyltransferase 3 (PRMT3) (Hs00411605_m1). Fold difference was calculated as 2ˆ(Ct((target gene) – Ct(B2M)) – (Ct(test sample) – Ct(calibrator sample))). The assays were repeated three times.

### Quantification and statistical analysis

Two tailed Student’s t-tests were performed in GraphPad Prism v9 (GraphPad Software) and the level of significance was defined to a *P* value less than 0.05. For the gene expression a two-way ANOVA with multiple correction was used comparing each data point to the expression of WT AIRE.

## Data Availability

• All data reported in this paper will be shared by the lead contact upon request. • This paper does not report original code.

## References

[1] Anderson M.S., Venanzi E.S., Chen Z., Berzins S.P., Benoist C., Mathis D. (2005). The cellular mechanism of Aire control of T cell tolerance. Immunity.

[2] Malchow S., Leventhal D.S., Lee V., Nishi S., Socci N.D., Savage P.A. (2016). Aire enforces immune tolerance by directing autoreactive T cells into the regulatory T cell lineage. Immunity.

[3] Husebye E.S., Anderson M.S., Kämpe O. (2018). Autoimmune polyendocrine syndromes. N. Engl. J. Med..

[4] Wolff A.S.B., Erichsen M.M., Meager A., Magitta N.F., Myhre A.G., Bollerslev J., Fougner K.J., Lima K., Knappskog P.M., Husebye E.S. (2007). Autoimmune polyendocrine syndrome type 1 in Norway: phenotypic variation, autoantibodies, and novel mutations in the autoimmune regulator gene. J. Clin. Endocrinol. Metab..

[5] Ricotta E.E., Ferré E.M.N., Schmitt M.M., DiMaggio T., Lionakis M.S. (2022). Prevalence of APECED-like clinical disease in an electronic Health record database, USA. J. Clin. Immunol..

[6] Meloni A., Furcas M., Cetani F., Marcocci C., Falorni A., Perniola R., Pura M., Bøe Wolff A.S., Husebye E.S., Lilic D. (2008). Autoantibodies against type I interferons as an additional diagnostic criterion for autoimmune polyendocrine syndrome type I. J. Clin. Endocrinol. Metab..

[7] Oftedal B.E., Hellesen A., Erichsen M.M., Bratland E., Vardi A., Perheentupa J., Kemp E.H., Fiskerstrand T., Viken M.K., Weetman A.P. (2015). Dominant mutations in the autoimmune regulator AIRE are associated with common organ-specific autoimmune diseases. Immunity.

[8] Goldfarb Y., Givony T., Kadouri N., Dobeš J., Peligero-Cruz C., Zalayat I., Damari G., Dassa B., Ben-Dor S., Gruper Y. (2021). Mechanistic dissection of dominant AIRE mutations in mouse models reveals AIRE autoregulation. J. Exp. Med..

[9] Eriksson D., Røyrvik E.C., Aranda-Guillén M., Berger A.H., Landegren N., Artaza H., Hallgren Å., Grytaas M.A., Ström S., Bratland E. (2021). GWAS for autoimmune Addison's disease identifies multiple risk loci and highlights AIRE in disease susceptibility. Nat. Commun..

[10] Laisk T., Lepamets M., Koel M., Abner E., Mägi R., Estonian Biobank Research Team (2021). Genome-wide association study identifies five risk loci for pernicious anemia. Nat. Commun..

[11] Chiou J., Geusz R.J., Okino M.L., Han J.Y., Miller M., Melton R., Beebe E., Benaglio P., Huang S., Korgaonkar K. (2021). Interpreting type 1 diabetes risk with genetics and single-cell epigenomics. Nature.

[12] Chascsa D.M., Ferré E.M.N., Hadjiyannis Y., Alao H., Natarajan M., Quinones M., Kleiner D.E., Simcox T.L., Chitsaz E., Rose S.R. (2021). APECED-associated hepatitis: clinical, biochemical, histological and treatment data from a large, predominantly American cohort. Hepatology.

[13] Gaetani M., Matafora V., Saare M., Spiliotopoulos D., Mollica L., Quilici G., Chignola F., Mannella V., Zucchelli C., Peterson P. (2012). AIRE-PHD fingers are structural hubs to maintain the integrity of chromatin-associated interactome. Nucleic Acids Res..

[14] Malchow S., Leventhal D.S., Nishi S., Fischer B.I., Shen L., Paner G.P., Amit A.S., Kang C., Geddes J.E., Allison J.P. (2013). Aire-dependent thymic development of tumor-associated regulatory T cells. Science.

[15] Sogkas G., Atschekzei F., Adriawan I.R., Dubrowinskaja N., Witte T., Schmidt R.E. (2021). Cellular and molecular mechanisms breaking immune tolerance in inborn errors of immunity. Cell. Mol. Immunol..

[16] Liston A., Farr A.G., Chen Z., Benoist C., Mathis D., Manley N.R., Rudensky A.Y. (2007). Lack of Foxp3 function and expression in the thymic epithelium. J. Exp. Med..

[17] Kreins A.Y., Bonfanti P., Davies E.G. (2021). Current and future therapeutic approaches for thymic stromal cell defects. Front. Immunol..

[18] Lemoine R., Pachlopnik-Schmid J., Farin H.F., Bigorgne A., Debré M., Sepulveda F., Héritier S., Lemale J., Talbotec C., Rieux-Laucat F. (2014). Immune deficiency-related enteropathy-lymphocytopenia-alopecia syndrome results from tetratricopeptide repeat domain 7A deficiency. J. Allergy Clin. Immunol..

[19] Pieplenbosch B., de Leijer J.H., van Dop W.A., Nagtegaal I.D., Witteman E.M. (2022). Thymoma-associated autoimmune enteropathy with colonic stricture: a diagnostic and histological challenge. Clin. J. Gastroenterol..

[20] Slavik T., Potgieter F.M., Brittain D. (2018). Thymoma-associated multiorgan autoimmunity with exclusive gastrointestinal tract involvement: case report and review of the literature. Virchows Arch..

[21] Marx A., Willcox N., Leite M.I., Chuang W.Y., Schalke B., Nix W., Ströbel P. (2010). Thymoma and paraneoplastic myasthenia gravis. Autoimmunity.

[22] Hapnes L., Willcox N., Oftedal B.E.V., Owe J.F., Gilhus N.E., Meager A., Husebye E.S., Wolff A.S.B. (2012). Radioligand-binding assay reveals distinct autoantibody preferences for type I interferons in APS I and myasthenia gravis subgroups. J. Clin. Immunol..

[23] Meager A., Wadhwa M., Dilger P., Bird C., Thorpe R., Newsom-Davis J., Willcox N. (2003). Anti-cytokine autoantibodies in autoimmunity: preponderance of neutralizing autoantibodies against interferon-alpha, interferon-omega and interleukin-12 in patients with thymoma and/or myasthenia gravis. Clin. Exp. Immunol..

[24] Garcia-Garcia A.B., Ivorra C., Martinez-Hervas S., Blesa S., Fuentes M.J., Puig O., Martín-de-Llano J.J., Carmena R., Real J.T., Chaves F.J. (2011). Reduced penetrance of autosomal dominant hypercholesterolemia in a high percentage of families: importance of genetic testing in the entire family. Atherosclerosis.

[25] Ammerlaan A.C.J., Ararou A., Houben M.P., Baas F., Tijssen C.C., Teepen J.L., Wesseling P., Hulsebos T.J.M. (2008). Long-term survival and transmission of INI1-mutation via nonpenetrant males in a family with rhabdoid tumour predisposition syndrome. Br. J. Cancer.

[26] Rosatelli M.C., Meloni A., Meloni A., Devoto M., Cao A., Scott H.S., Peterson P., Heino M., Krohn K.J., Nagamine K. (1998). A common mutation in Sardinian autoimmune polyendocrinopathy-candidiasis-ectodermal dystrophy patients. Hum. Genet..

[27] Zlotogora J., Shapiro M.S. (1992). Polyglandular autoimmune syndrome type I among Iranian Jews. J. Med. Genet..

[28] Bruserud Ø., Oftedal B.E., Landegren N., Erichsen M.M., Bratland E., Lima K., Jørgensen A.P., Myhre A.G., Svartberg J., Fougner K.J. (2016). A longitudinal follow-up of autoimmune polyendocrine syndrome type 1. J. Clin. Endocrinol. Metab..

[29] Ferre E.M.N., Rose S.R., Rosenzweig S.D., Burbelo P.D., Romito K.R., Niemela J.E., Rosen L.B., Break T.J., Gu W., Hunsberger S. (2016). Redefined clinical features and diagnostic criteria in autoimmune polyendocrinopathy-candidiasis-ectodermal dystrophy. JCI Insight.

[30] Nagamine K., Peterson P., Scott H.S., Kudoh J., Minoshima S., Heino M., Krohn K.J., Lalioti M.D., Mullis P.E., Antonarakis S.E. (1997). Positional cloning of the APECED gene. Nat. Genet..

[31] Pearce S.H., Cheetham T., Imrie H., Vaidya B., Barnes N.D., Bilous R.W., Carr D., Meeran K., Shaw N.J., Smith C.S. (1998). A common and recurrent 13-bp deletion in the autoimmune regulator gene in British kindreds with autoimmune polyendocrinopathy type 1. Am. J. Hum. Genet..

[32] Zaidi G., Bhatia V., Sahoo S.K., Sarangi A.N., Bharti N., Zhang L., Yu L., Eriksson D., Bensing S., Kämpe O. (2017). Autoimmune polyendocrine syndrome type 1 in an Indian cohort: a longitudinal study. Endocr. Connect..

[33] Heino M., Peterson P., Kudoh J., Shimizu N., Antonarakis S.E., Scott H.S., Krohn K. (2001). APECED mutations in the autoimmune regulator (AIRE) gene. Hum. Mutat..

[34] Garelli S., Dalla Costa M., Sabbadin C., Barollo S., Rubin B., Scarpa R., Masiero S., Fierabracci A., Bizzarri C., Crinò A. (2021). Autoimmune polyendocrine syndrome type 1: an Italian survey on 158 patients. J. Endocrinol. Invest..

[35] Landegren N., Sharon D., Shum A.K., Khan I.S., Fasano K.J., Hallgren Å., Kampf C., Freyhult E., Ardesjö-Lundgren B., Alimohammadi M. (2015). Transglutaminase 4 as a prostate autoantigen in male subfertility. Sci. Transl. Med..

[36] Alimohammadi M., Dubois N., Sköldberg F., Hallgren A., Tardivel I., Hedstrand H., Haavik J., Husebye E.S., Gustafsson J., Rorsman F. (2009). Pulmonary autoimmunity as a feature of autoimmune polyendocrine syndrome type 1 and identification of KCNRG as a bronchial autoantigen. Proc. Natl. Acad. Sci. USA.

[37] Ekwall O., Hedstrand H., Haavik J., Perheentupa J., Betterle C., Gustafsson J., Husebye E., Rorsman F., Kämpe O. (2000). Pteridin-dependent hydroxylases as autoantigens in autoimmune polyendocrine syndrome type I. J. Clin. Endocrinol. Metab..

[38] Ahonen P., Myllärniemi S., Sipilä I., Perheentupa J. (1990). Clinical variation of autoimmune polyendocrinopathy-candidiasis-ectodermal dystrophy (APECED) in a series of 68 patients. N. Engl. J. Med..

[39] Fernández E., Vicente A., Zapata A., Brera B., Lozano J.J., Martínez C., Toribio M.L. (1994). Establishment and characterization of cloned human thymic epithelial cell lines. Analysis of adhesion molecule expression and cytokine production. Blood.

[40] Koh A.S., Kuo A.J., Park S.Y., Cheung P., Abramson J., Bua D., Carney D., Shoelson S.E., Gozani O., Kingston R.E. (2008). Aire employs a histone-binding module to mediate immunological tolerance, linking chromatin regulation with organ-specific autoimmunity. Proc. Natl. Acad. Sci. USA.

