# Supplementary material for: Dominant-negative heterozygous mutations in AIRE confer diverse autoimmune phenotypes
Source: iScience. 2023 May 5;26(6):106818. doi: 10.1016/j.isci.2023.106818 (PMC10206195; doi:10.1016/j.isci.2023.106818)
Supplement: Document S1. Figure S1 and Table S1 [file mmc1.pdf]

iScience, Volume *26*

Supplemental information

Dominant-negative heterozygous mutations

in AIRE confer diverse autoimmune phenotypes

Bergithe E. Oftedal, Kristian Assing, Safa Baris, Stephanie L. Safgren, Isik S. Johansen, Marianne Antonius Jakobsen, Dusica Babovic-Vuksanovic, Katherine Agre, Eric W. Klee, Emina Majcic, Elise M.N. Ferré, Monica M. Schmitt, Tom DiMaggio, Lindsey B. Rosen, Muhammad Obaidur Rahman, Dionisios Chrysis, Aristeidis Giannakopoulos, Maria Tallon Garcia, Luis Ignacio González-Granado, Katherine Stanley, Jessica Galant-Swafford, Pim Suwannarat, Isabelle Meyts, Michail S. Lionakis, and Eystein S. Husebye

Supplemental table 1. ClinVar description for the different AIRE variants, related to Table 3.

Mutation	ClinVar description
c.901G>A	The V301M variant in the AIRE gene has been reported previously in the heterozygous state in a patient with Addison's disease and autoimmune thyroiditis, in a patient with autoimmune polyendocrinopathy-candidiasis-ectodermal dystrophy, and in a patient with systemic sclerosis and autoimmune thyroiditis (Soderbergh et al., 2000; Ferrera et al., 2007; Orlova et al., 2010). These reported heterozygous cases have prompted the inclusion of V301M in several in vitro studies of AIRE, many of which have failed to show a conclusive deleterious effect for V301M (Bottomly, 2005; Chakravarty, 2009). One study did demonstrate that V301M affects expression of AIRE target genes by reducing the chromatin level interaction with its protein partners involved in transcriptional activation (Gaetani et al., 2012). The V301M variant is observed in 153/25596 (0.60%) alleles from individuals of Finnish European background, including 2 homozygous individuals, in large population cohorts (Lek et al., 2016). The V301M variant is a conservative amino acid substitution, which occurs at a position in the PHD-type 1 zinc finger domain where amino acids with similar properties to Valine are tolerated across species. We interpret V301M as a variant of uncertain significance.
c.916G>A	In silico analysis supports that this missense variant has a deleterious effect on protein structure/function; Has not been previously published as pathogenic or benign to our knowledge
c.926T>C	
c.977C>T	This sequence change replaces proline, which is neutral and non-polar, with leucine, which is neutral and non-polar, at codon 326 of the AIRE protein (p.Pro326Leu). This variant is present in population databases (rs179363885, gnomAD 0.003%). This missense change has been observed in individual(s) with AIRE-related conditions (PMID: 11275943, 28911151, 29666621). In at least one individual the data is consistent with the variant being in trans (on the opposite chromosome) from a pathogenic variant. It has also been observed to segregate with disease in related individuals. ClinVar contains an entry for this variant (Variation ID: 68232). Advanced modeling of protein sequence and biophysical properties (such as structural, functional, and spatial information, amino acid conservation, physicochemical variation, residue mobility, and thermodynamic stability) performed at Invitae indicates that this missense variant is expected to disrupt AIRE protein function.

	Experimental studies have shown that this missense change affects AIRE function (PMID: 26084028). For these reasons, this variant has been classified as Pathogenic. (less)
c.982C>T	This sequence change replaces arginine with tryptophan at codon 328 of the AIRE protein (p.Arg328Trp). The arginine residue is weakly conserved and there is a moderate physicochemical difference between arginine and tryptophan. This variant has not been reported in the literature in individuals with AIRE-related conditions. Algorithms developed to predict the effect of missense changes on protein structure and function are either unavailable or do not agree on the potential impact of this missense change (SIFT: "Deleterious"; PolyPhen-2: "Probably Damaging"; Align-GVGD: "Class C0"). In summary, the available evidence is currently insufficient to determine the role of this variant in disease. Therefore, it has been classified as a Variant of Uncertain Significance.
c.1102C>G	This sequence change replaces proline with alanine at codon 368 of the AIRE protein (p.Pro368Ala). The proline residue is weakly conserved and there is a small physicochemical difference between proline and alanine. This variant is not present in population databases (ExAC no frequency). This variant has not been reported in the literature in individuals with AIRE-related conditions. Algorithms developed to predict the effect of missense changes on protein structure and function (SIFT, PolyPhen-2, Align-GVGD) all suggest that this variant is likely to be tolerated, but these predictions have not been confirmed by published functional studies and their clinical significance is uncertain. In summary, the available evidence is currently insufficient to determine the role of this variant in disease. Therefore, it has been classified as a Variant of Uncertain Significance.
c.1399G>C	This sequence change replaces glycine with arginine at codon 467 of the AIRE protein (p.Gly467Arg). The glycine residue is moderately conserved and there is a moderate physicochemical difference between glycine and arginine. This variant is present in population databases (rs202018301, ExAC 0.01%). This variant has not been reported in the literature in individuals with AIRE-related disease. Algorithms developed to predict the effect of missense changes on protein structure and function are either unavailable or do not agree on the potential impact of this missense change (SIFT: "Deleterious"; PolyPhen-2: "Possibly Damaging"; Align-GVGD: "Class C0"). Algorithms developed to predict the effect of sequence changes on RNA splicing suggest that this variant may create or strengthen a splice site, but this prediction has not been confirmed by published transcriptional studies. In summary, the available evidence is currently insufficient to determine the role of this variant in disease. Therefore, it has been classified as a Variant of Uncertain Significance

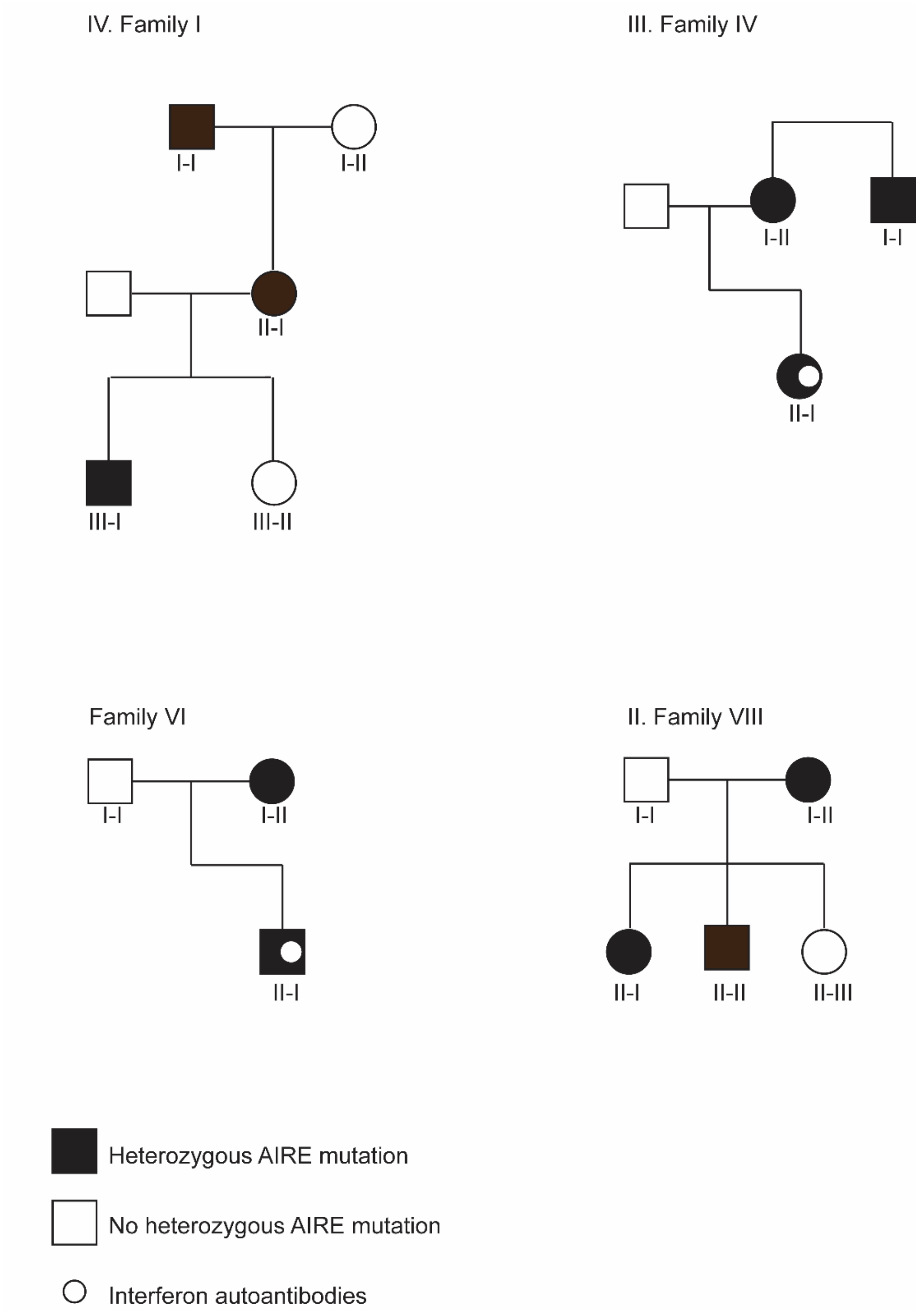

Supplemental Figure 1. Pedigree overview of the different families, related to Table 4. The families described in Table 4 are shown, with the carriers of heterozygous AIRE mutations in black, and the presence of autoantibodies against interferons as white circles.
